# Minimal Residual Disease in Multiple Myeloma: State of the Art and Future Perspectives

**DOI:** 10.3390/jcm9072142

**Published:** 2020-07-07

**Authors:** Roberto Mina, Stefania Oliva, Mario Boccadoro

**Affiliations:** Myeloma Unit, Division of Hematology, University of Torino, Azienda Ospedaliero-Universitaria Città della Salute e della Scienza di Torino, via Genova 3, 10126 Torino, Italy; stefania.olivamolinet@gmail.com (S.O.); mario.boccadoro@unito.it (M.B.)

**Keywords:** multiple myeloma, minimal residual disease, next-generation sequencing, next-generation flow

## Abstract

Minimal residual disease (MRD) detection represents a sensitive tool to appropriately measure the response obtained with therapies for multiple myeloma (MM). The achievement of MRD negativity has superseded the conventional complete response (CR) and has been proposed as a surrogate endpoint for progression-free survival and overall survival. Several techniques are available for the detection of MRD inside (next-generation sequencing, flow cytometry) and outside (PET/CT, magnetic resonance) the bone marrow, and their complementary use allows a precise definition of the efficacy of anti-myeloma treatments. This review summarizes MRD data and results from previous clinical trials, highlights open issues related to the role of MRD in MM and discusses how MRD could be implemented in clinical practice to inform on patient prognosis and drive therapeutic decisions.

## 1. Introduction

In the last two decades, the treatment approach to multiple myeloma (MM) has been redefined by the development of a number of active compounds of several drug-classes. While only few patients were able to achieve a complete remission (CR) with conventional chemotherapy, approximately half of treated patients achieved a CR with multi-drug regimens based on non-cross resistant anti-MM agents such as immunomodulatory drugs (IMiDs), proteasome inhibitors (PIs) and monoclonal antibodies (mAbs), with or without high-dose melphalan (HDM) and autologous stem-cell transplantation (ASCT) [1,2,3,4,5,6].

As compared to non-CR patients, the achievement of a CR has been associated to significantly longer progression-free survival (PFS) and overall survival (OS) [7,8]. Even in CR patients, however, treatment intensification with HDM-ASCT and maintenance therapy were associated to better survival outcomes [9,10,11].

It is now clear that the real prognostic value of CR relies on the absence of minimal residual disease (MRD). Since in 2016 the International Myeloma Working Group (IMWG) updated the response criteria by including new response categories based on the assessment of MRD both inside and outside the bone marrow [12], interest has emerged in MRD as a surrogate endpoint for survival outcomes and in its incorporation in clinical trials.

In this review, we discuss how MRD assessment is currently being performed, what previous studies have taught us about the role of MRD in the treatment of MM, and how MRD could change the management of MM patients in the next future.

## 2. How Can Minimal Residual Disease Be Assessed?

### 2.1. MRD in the Bone Marrow

As per IMWG new response criteria [12], MRD should first be assessed in the bone marrow. Two different techniques, next-generation sequencing (NGS) and multiparameter flow cytometry (MFC), have been largely studied in the last 20 years, and their different characteristics are showed in Table 1.

Molecular biology has been a matter of debate for a long time, until the implementation of NGS allowed to consider MRD as a novel primary endpoint in several ongoing clinical trials. Currently, NGS surpassed allele-specific oligonucleotides quantitative polymerase chain reaction (ASO-qPCR), since NGS does not need patient-specific primers and probes and showed higher applicability due to a better marker identification rate at diagnosis (90–92% with NGS vs. 50–60% with ASO-qPCR), thus overcoming the failure to detect clonality by ASO-qPCR after IGH somatic hypermutation [14]. 

The most commonly adopted NGS approach (Clonoseq^®^, Adaptive Biotechnologies, Seattle, US-WA) allowed the achievement of high sensitivity levels (up to 1E-06) in several clinical trials [15].

In 2014, Martinez-Lopez et al. have first showed the prognostic value of deep sequencing and its good concordance with MFC and ASO-PCR (83% and 85%, respectively). Specifically, patients who were MRD negative by NGS had a significantly longer time to progression (TTP) and overall survival (OS), as compared with MRD-positive patients. In patients in CR, the TTP was significantly longer in MRD-negative than in MRD-positive patients (131 vs. 35 months; *P* = 0.0009) [16]. Perrot et al. have confirmed these important findings in a larger series of MM patients enrolled in the Intergroupe Francophone du Myélome (IFM) 2009 trial, in which progression-free survival (PFS) was significantly prolonged in MRD-negative vs. MRD-positive patients both at pre- and post-maintenance timepoints [17]. Other non-commercial NGS technologies are under investigation: the LymphoTrack^®^ assay (Invitrogen, US-MA) has been recently validated in a phase II study [18], and the EuroClonality-NGS Consortium (an international group of 21 academic laboratories experienced in NGS) has recently validated IG/TR NGS assays and a bioinformatic tool for an academic study on MRD [19]. 

Flow cytometry is able to distinguish normal monoclonal plasma cells from aberrant ones by detecting high or low expression of cell-surface markers and monoclonal expression of intra-cytoplasmic markers (immunoglobulin light chain) [20]. Historically, 4- to 7-color flow cytometry assays were used for MRD detection and showed a strong correlation with both PFS and OS [21]. 

Advanced 8-color 2-tube or 10-color 1-tube assays (next-generation flow, NGF) have now superseded older techniques. The 10-color 2-tube NGF EuroFlow™ showed a higher sensitivity vs. conventional 8-color flow-MRD: 25% of patients who were classified as MRD negative by conventional 8-color flow-MRD were classified as MRD positive by NGF [22]. In a large cohort of MM patients, Paiva et al. showed that MRD by NGF has a high applicability (99%) and a high prediction accuracy of both PFS and OS: only 7% of MRD-negative patients (sensitivity 10^−6^) relapsed, most of them with extramedullary disease. Paiva et al. also nicely discussed the reasons for such a high sensitivity: (1) the evaluation of B-cell precursors, mast cells and nucleated red blood cells by using a standardized approach could detect hemodiluted samples that were considered inadequate for MRD assessment; (2) a high number of nucleated cells was acquired (~10 millions); (3) the use of the automatic population separator eliminated the operator-dependent variability [22,23]. 

Ongoing clinical trials are evaluating NGS vs. MFC/NGF and their correlation. The CASSIOPEIA trial reported a good concordance between NGS and NGF in ≥CR patients (83.5% in paired samples, sensitivity of 10^−5^) [24]. In the FORTE study, NGS was compared to second-generation MFC (both at a sensitivity of 10^−5^) in ≥CR patients and revealed an observed agreement rate of 86%. In all but one of these discordances, MRD positivity was not detected using MFC [25].

### 2.2. MRD Outside the Bone Marrow

While imaging plays a vital role in the diagnosis of MM, its role in the response assessment to anti-MM treatments is emerging, also in consideration of the spatial heterogeneity of myeloma conferred by the patchy infiltration of bone marrow plasma cells and the potential presence of extramedullary disease [26,27]. In this regard, whole body imaging with positron emission tomography and computed tomography (PET/CT) or magnetic resonance imaging (MRI) provide important complementary information about residual disease after therapy.

18Fluorine-fluoro-deoxyglucose (18F-FDG) PET/CT is currently considered the gold standard for evaluating and monitoring the metabolic response to therapy [28,29]. In an ongoing effort to standardize standardized uptake value (SUV) cut-offs in MM patients, the Deauville scores [30] proved to be applicable and representative of patients’ outcomes, identifying the liver background (Deauville score 4) as the best reference for the definition of a PET-complete metabolic response [13].

However, approximately 10–15% of patients with active MM may have a false-negative PET/CT result, since the lack of hexokinase enzyme reduces the 18F-FDG avidity of plasma cells. This limits the applicability of FDG-PET/CT in MM [31] and new PET/CT tracers targeting different metabolic pathways or receptors expressed by MM cells and acting as molecular imaging biomarkers are currently being investigated in clinical trials [32,33]. 

PET/CT has a prognostic value in MM: in patients achieving a CR, FDG-PET/CT negativity after ASCT predicted a lower risk of progression or death, as compared to patients with metabolically active lesions. Different studies also confirmed the complementarity of PET/CT and bone marrow techniques [34,35]. Rasche et al. showed that patients who were both Flow-MRD negative and PET/CT negative had the best PFS outcome, as compared to patients who were Flow-MRD negative but PET/CT positive or vice-versa. Paiva et al. demonstrated that, despite a long median PFS, a proportion of NGF-negative patients relapsed with extramedullary disease [23]. In the CASSIOPEIA study [36], a low agreement between bone marrow MRD techniques and PET/CT were reported. These observations confirmed the importance of combining bone marrow and imaging techniques to fully evaluate MRD in MM.

MRI is a non-invasive radiological technique that can detect both MM bone involvement, with the ability to describe the infiltration pattern by plasma cells (normal, focal, diffuse, heterogeneous), and the presence of extramedullary disease. In older studies, traditional MRI has been shown to be more sensitive and specific than FDG-PET/CT for the detection of focal lesions and diffuse infiltration [37,38]. However, focal lesions may take time to disappear even in responding patients, thus making traditional MRI a less desirable tool for MRD assessment as compared to FDG-PET/CT. The predictive value of the disappearance of MM lesions detected by MRI and FDG-PET/CT in terms of PFS and OS was assessed in a prospective study [34]. While the disappearance of positive lesions detected by FDG-PET/CT predicted better PFS/OS as compared to those of patients with persistent positive lesions, this was not true for patients assessed by traditional MRI. Diffusion-weighted MRI (DW-MRI) is a functional technique that provides a quantitative assessment of whole-body tumor burden through the use of the apparent diffusion coefficient (ADC) [39]. In two retrospective studies comparing DW-MRI and FDG-PET/CT for the assessment of bone infiltration in MM patients, DW-MRI showed higher sensitivity in detecting both diffuse infiltration and focal lesions as compared to FDG-PET/CT [40,41]. This new functional technique may challenge the role of FDG-PET/CT in evaluating MRD outside the bone marrow, particularly in light of the percentage of false-negative patients due to a low-expression of hexokinase-2 [31].

The use of whole-body imaging techniques to evaluate the presence of MM cells is of uttermost importance, since MM is characterized by a potential patchy infiltration of the bone marrow by plasma cells as well as by the presence of extramedullary disease [42]. Furthermore, clonal heterogeneity may characterize MM, with the coexistence, in the same patient, of multiple clones with different molecular and biological features [27,43]. In this light, the use of both “inside” (NGF, NGS) and “outside” (MRI, PET/CT) bone marrow techniques allows the monitoring of MRD in a complementary, rather than alternative, fashion.

The use of peripheral blood could be an attractive method for MRD detection that overcomes the site-dependence and invasiveness of repetitive bone marrow aspirations, also tackling the spatial heterogeneity of MM. In this view, liquid biopsy, which can detect circulating tumor DNA and circulating tumor cells, would be a promising option. 

However, some controversial results have been reported about the concordance between peripheral blood and bone marrow assessments in terms of MRD results. Available data showed a high concordance in clonal mutations between circulating tumor cells and bone marrow paired samples, with some subclonal mutations being found exclusively in circulating tumor cells [44,45,46]. The NGF assay for MRD evaluation is also feasible and highly sensitive for the detection and enumeration of circulating tumor cells in MM, even though a significant proportion of MM cases that are positive in the bone marrow or at serum immunofixation still had undetectable circulating tumor cells in paired blood samples (40 and 30%, respectively) [47]. Hence, based on current data, both circulating tumor DNA and circulating tumor cells may be used for mirroring the genetic landscape of bone marrow-based disease and recapitulate efficiently the spatial intra-subclonal heterogeneity (particularly for those patients with extramedullary disease), but efforts are needed to improve standardization and increase their sensitivity in order to replace bone marrow MRD assessment. 

An alternative serological method, the quantitative immunoprecipitation mass spectrometry (QIP-MS), was developed. This polyclonal antibody-based technology identifies intact immunoglobulins at a higher sensitivity when compared to standard SPEP, with a moderate concordance with bone marrow NGF evaluation, thus offering a new way to detect MRD in peripheral blood for MM patients [48]. The MALDI TOF technique has recently showed higher sensitivity than standard SPEP and good concordance with bone marrow flow-MRD results (62%) [49].

## 3. Current Evidence on the Role of MRD Assessment in MM

Several studies support the use of MRD for response monitoring in MM (Table 2). The positive correlation between MRD negativity and prolonged PFS and OS was shown by two meta-analyses including several clinical trials in which MRD was assessed with a sensitivity level of 10^−4^–10^−5^ by both immunophenotypic and molecular techniques [21,50]. 

The evidence that MRD status stratified patients in CR into 2 groups (CR-MRD negative and CR-MRD positive, the former having significantly longer PFS and OS) led to the idea that “conventional” CR was no more a clinically meaningful endpoint. Lahuerta and colleagues demonstrated that MRD-negative patients by MFC at a sensitivity of 10^−4^–10^−5^ in a conventionally defined CR had better PFS and OS as compared to MRD-positive CR patients. They also showed that the patients in the latter group had similar outcomes to patients in PR [61]. In light of this observation, MRD negativity superseded the former CR definition as an endpoint, and the latest clinical trials have already incorporated the MRD negativity rate as a clinical endpoint.

As reported in other hematologic diseases, also in MM the higher the sensitivity for the definition of MRD undetectability, the better is the outcome. Both NGS and NGF confirmed this result: patients who achieved MRD negativity at a sensitivity of 10^−6^ showed a prolonged PFS, as compared to those who were MRD negative at 10^−5^ or higher [17,23]. 

Whether MRD negativity could abrogate the high risk associated with unfavorable cytogenetic abnormalities detected by fluorescence in situ hybridization (FISH) or biological characteristics such as the International Staging System (ISS) is still a matter of debate. The achievement of MRD negativity, as well as of sustained MRD negativity, is less frequent in high-risk patients than in standard-risk patients. In the IFM 2009 and EMN02 trials, del(17p)-positive patients achieved MRD negativity in 11% and 7% of cases, respectively [17,52]. However, it was reported that high-risk patients (defined by ISS, Revised ISS [R-ISS] and FISH) who reached MRD negativity had a survival rate comparable to that of standard-risk patients. Paiva et al. showed that the TTP in MRD-negative patients was similar irrespective of FISH status (not reached; P = 0.70), whereas FISH status still had a significant impact on TTP in MRD-positive patients (standard risk, median TTP 15 months vs. 12 months for MRD-positive high-risk patients; P = 0.02) [51]. The EMN02 study confirmed this evidence: patients with high-risk MM at diagnosis (defined by ISS and FISH) and persistent residual disease after treatment had a dismal outcome (median PFS of 7 months for patients with ISS stage III and 15 months for those with high risk by FISH) [52]. Similarly, Paiva et al. showed that the initial R-ISS prognostic stratification was meaningful only in patients with persistent MRD, but not in MRD-negative patients [23]. 

Many studies demonstrated that the favorable prognosis associated with MRD negativity is treatment-naïve. Perrot et al. observed no differences in terms of PFS for MRD-negative patients who received HDM/ASCT vs. those who did not receive ASCT [17]; however, a higher number of patients in the ASCT group obtained MRD negativity, as compared to the non-ASCT group (79% vs. 65%, P < 0.001) [62]. In transplant-ineligible patients, the addition of daratumumab to the standard combinations bortezomib-melphalan-prednisone and lenalidomide-dexamethasone increased the rates of MRD negativity in comparison with standard therapy (27–24% vs. 7%, respectively; P < 0.001 for both) and prolonged PFS and OS in the overall population. Again, the PFS was similar in MRD-negative patients, irrespectively of previous treatment [1,2,3].

In the phase III CASSIOPEIA study, the addition of daratumumab to the standard induction regimen bortezomib-thalidomide-dexamethasone (D-VTd) induced significantly higher rates of MRD at 10^−5^ than VTd alone, with a PFS benefit in patients achieving MRD negativity [6]. Interestingly, the addition of daratumumab not only prolonged the PFS of MRD-positive patients but also seemed to benefit MRD-negative patients [6]. A longer follow-up is needed to confirm these data.

The achievement of MRD negativity is a treatment goal that could be pursued not only in young, transplant-eligible patients, but also in older ones. This was confirmed by the Spanish PETHEMA/GEM2010MAS65 study, in which age did not negatively affect the outcome of MRD-negative patients (median TTP was not reached for patients aged 65–75 years vs. >75 years; P = 0.74) [51]. 

## 4. Incorporation of MRD Results into Clinical Practice

Despite its incorporation into the IMWG response criteria, there is no current evidence that MRD can be used to drive therapeutic choices and tailor patient treatment in standard clinical practice. Nonetheless, MRD has been recently adopted as a new primary endpoint in ongoing trials, which will inform whether MRD status might be used to modulate treatment strategies.

High-dose melphalan plus ASCT is a standard intensification strategy for the treatment of newly diagnosed (ND)MM patients younger than 70–75 years of age [63]. The role of ASCT has been frequently challenged in the past, but all studies reported a PFS advantage for patients receiving ASCT, as compared to no ASCT, although with an inconsistent OS benefit [62,64,65,66]. Whether MRD status could be used to decide between a transplant-based or a non-transplant-based approach in an era of highly effective induction regimens is still a matter of debate. In the IFM 2009 study, no difference in terms of PFS was found in MRD-negative patients irrespective of treatment received (ASCT or VRD), although a higher number of patients in the ASCT arm were MRD negative as compared to the VRD arm (30% vs. 21%) [17]. In the FORTE study, the rate of MRD negativity (10^−5^, by MFC) was similar (58% vs. 54%) in patients who received 4 induction cycles with carfilzomib-lenalidomide-dexamethasone (KRd) followed by ASCT and further 4 KRd consolidation cycles vs. 12 KRd cycles without ASCT [56]. 

In a recently presented study, 4 daratumumab-KRd (D-KRd) induction cycles succeeded in converting to MRD negativity (10^−5^, by NGS) 40% of treated patients. After HDM-ASCT, the MRD negativity rate increased up to 73% [59], a proportion similar to that observed in another study presented at the ASH 2019 meeting by Landgren et al. [60], in which 77% of patients who received 8 induction cycles of D-KRd without transplant were MRD negative (10^−5^, by NGS). With the development of new multi-drug regimens, the benefit in terms of response rates conferred by HDM plus ASCT over no ASCT seemed to decrease, although a long-term follow-up is needed to determine potential differences in terms of PFS and OS. Altogether, these data suggest the hypothesis that patients who are able to achieve MRD negativity with the induction therapy may not need HDM-ASCT, thus supporting the development of controlled trials randomizing patients to ASCT vs. non-transplant-based strategies. In this light, results from the FORTE study will help determine the future of HDM-ASCT in the management of young MM patients. 

In the context of the therapeutic approach to MM, another open question concerns the need for maintenance therapy after induction/HDM-ASCT in MRD-negative patients and its optimal duration. Since MRD negativity at day + 100 after ASCT is associated with prolonged survival [67], it is legitimate to speculate that MRD-negative patients may not need maintenance therapy after ASCT, thus benefiting from a treatment-free interval. In the Myeloma XI study, the best outcome in terms of PFS was reported in MRD-negative patients who still received lenalidomide maintenance. Previous studies also showed that lenalidomide maintenance can convert a significant number of MRD-positive patients to MRD negativity (44% in the EMN02 study, 32% In the Myeloma XI study) [52,68]. On the basis of the data generated so far, it is not yet possible to advise against maintenance therapy for patients who are MRD negative after ASCT or to encourage treatment discontinuation for those patients who become MRD negative during maintenance. Results of ongoing clinical trials specifically designed to address these points are eagerly awaited. In the EMN17 Perseus trial (NCT03710603), patients with sustained MRD negativity (at least 2 negative samples 12 months apart) who receive maintenance treatment with daratumumab-lenalidomide have the opportunity to withhold daratumumab maintenance after at least 2 years and continue with lenalidomide only. In the SWOG S1803 (NCT04071457) study comparing daratumumab-lenalidomide to lenalidomide alone as maintenance after ASCT, patients who are MRD negative after 2 years of maintenance are randomized to treatment discontinuation vs. continuation, thus allowing the investigators to address this important issue.

So far, the exact frequency of MRD testing is unknown. MRD monitoring could provide clinically useful information not only because, as previously reported, MRD-positive patients can be converted to MRD-negativity during treatment but also because MRD-negative patients can become MRD positive over time, this being an early sign of relapsing MM. An analysis of MRD kinetics with serial MRD assessments (every 6 months) during lenalidomide maintenance showed that MRD reappearance from a previous MRD-negative sample predicted 4 months in advance a biochemical relapse (i.e., the reappearance of a monoclonal component in serum or urine) and 9 months in advance a clinical relapse [69]. In the same study, however, 30% of patients with persistent MRD positivity did not experience any relapse. From a clinical perspective, these data could help clinicians restart treatment before the occurrence of clinical relapse, thus preventing the morbidity associated to MM proliferation [70]. Altogether, this evidence prompts the development of further studies to explore the role of MRD assessment and kinetics and their correlation with survival outcomes. 

Finally, given that MRD negativity is a strong prognostic factor for PFS and OS, another important open question that needs to be clarified by randomized trials is whether patients who are MRD positive at a specific time point—such as after HDM-ASCT or consolidation—would benefit from a treatment switch to non-cross-resistant drugs or from treatment intensification, in order to maximize the odds of achieving MRD negativity, particularly in light of the promising results obtained with immunotherapeutic strategies tested in the relapse setting. 

Aside from the upfront setting, where the odds of achieving CR and MRD negativity are higher, MRD data also came from the relapse setting, in which the latest studies, in particular with antibodies and cellular products, reported unprecedented rates of CR and even of MRD negativity among heavily pretreated patients (Table 3). The rate of MRD-negative patients (10^−5^) treated at relapse with an anti-CD38 mAb (either daratumumab or isatuximab) in combination with IMiDs or PIs ranged between 5% and 30% [71,72]. In a study on the CAR T-cell therapy bb2121, Raje et al. observed an impressive 94% of MRD negativity (NGS, 10^−5^) among evaluable, heavily pre-treated RRMM patients. Despite this outstanding result, the median PFS of the study was only 12 months, with many MRD-negative patients who relapsed despite the achievement of a deep response [73]. Although newer agents allow relapsed patients to obtain deep responses that are ultimately associated with prolonged survival, these data suggest that we still need to understand the value of MRD at relapse and how to comprehensively assess disease response in this setting.

## 5. Conclusions

Several studies demonstrated that MRD is a strong and reliable prognostic factor in terms of relapse and survival of MM patients, and efforts are ongoing to validate MRD assessment as a surrogate endpoint, in order to accelerate the interpretation of clinical trial results. However, a number of open questions—concerning the optimal technique(s) for MRD assessment, its availability worldwide, the timing and monitoring frequency and its ability to modify the treatment pathway—suggest that MRD testing is not yet ready to enter the clinical practice and impact treatment strategies. MRD-based clinical trials and their results are eagerly needed to fully understand the value of MRD testing in MM and to demonstrate the validity of using MRD as the main driver of clinical decisions, both in the upfront and relapsed settings.

## Figures and Tables

**Table 1:** MRD techniques for myeloma recommended by the IMWG [12]: pros and cons.

	Next-Generation Sequencing (NGS)	Next-Generation Flow (NGF)	Imaging (PET/CT)
**Availability**	Adaptive Biotechnologies (Seattle, US-WA); commercial service; FDA approved; academic platforms ongoing	Worldwide	Almost all hematological centers
**Applicability**	90–92%	Roughly 100%	85–90%
**Baseline assessment**	Required for identification of dominant clonotype	Not required	Required for identification of focal lesions or extramedullary disease
**Processing** **requirements**	Fresh sample is not required; both fresh and stored samples	Fresh samples are required; assessment within 24–36 h	NA
**Standardization**	Yes; Adaptive Biotechnologies (Seattle, US-WA)	Yes; EuroFlow Consortium	Ongoing [13]
**Sample quality control**	Evaluable by global bone marrow cell analysis	Not possible	NA
**Quantitative**	Yes	Yes	Yes
**Sensitivity**	1 in 10^−5^–10^−6^	1 in 10^−5^–10^−6^	Spatial resolution limit of 5 mm for focal lesions
**Turnaround and complexity**	1–2 weeks; bioinformatic support required	3–4 h; flow cytometry skills required; automated software available	80–90 min for the procedure; 30 min for analysis. Requires nuclear medicine support
**Clonal evolution**	Evaluable by tracking minor clonotypes	Not evaluable	Evaluable by focal lesion biopsies
**Patchy disease evaluation**	No	No	Yes
**Costs**	Roughly 1500 USD/sample	Roughly 300 USD/sample	Roughly 1350 USD/patient

**Abbreviations.** IMWG, International Myeloma Working Group; NGS, next-generation sequencing; NGF, next-generation flow; PET/CT, positron emission tomography/computed tomography; FDA, Food and Drug Administration; NA, not available; h, hours; min, minutes.

**Table 2:** NDMM patients treated with novel agents: latest selected studies with MRD analysis.

Reference	MRD Technique (Sensitivity)	Study Population and Treatment	Time Point Assessment	MRD Rate	Survival Outcomes
Paiva B. et al. 2016 [51]	MFC (10^−5^)	NTE NDMM pts (N = 162) Sequential or alternating VMP/Rd cycles	With response ≥VGPR after 9 or 18 sequential or alternating VMP/Rd cycles	Sequential arm: 9-cycles: 20%; 18-cycles: 46% Alternating arm: 9-cycles: 19%; 18-cycles: 33%	Median TTP: NR vs. 15 mo
Oliva S. et al. 2017 [52]	MFC (10^−5^)	TE NDMM pts (N = 316) VCd induction, VMP vs. ASCT intensification, VRd vs. no consolidation followed by lenalidomide maintenance	With response ≥VGPR; pre and during maintenance	Post consolidation: 76%	3-year PFS: 77% vs. 50%
Ocio E.M. et al. 2018 [53]	NGF (10^−5^) and NGS (10^−5^)	NTE NDMM pts (N = 16) Isa-VRd induction followed by Isa-Rd maintenance	Longitudinal	NGF 44% (18% at 10^−6^) NGS 50% (33% at 10^−6^)	NA
Zimmerman T. et al. 2018 [54]	MFC (10^−4^−10^−5^) and NGS (10^−6^)	TE NDMM pts (N = 76) 4 cycles of KRd induction-ASCT-4 cycles of KRd consolidation and 10 cycles of KRd extended consolidation	Longitudinal	MFC: post consolidation (cycle 8): 82%; post extended consolidation (cycle 18): 90% NGS: post consolidation (cycle 8): 66%; post extended consolidation (cycle 18): 71%	According to cycle 8, MRD status by MFC and/or NGS: 2-year PFS 100% vs. 93%
Perrot A. et al. 2018 [17]	NGS (10^−6^)	TE NDMM pts (N = 509) 8 VRd cycles or 3 VRd + ASCT + 2 VRd cycles followed by lenalidomide maintenance	Pre or post maintenance	VRd alone arm: 20% ASCT arm: 30%	Median PFS: NR vs. 29 months
Mateos M.V. et al. 2019 [2,3,55]	NGS (10^−5^)	NTE NDMM pts (N = 706) - Dara-VMp vs.- VMp arm	Longitudinal	Dara-VMp arm: 28% VMp arm: 7%	NA
Facon T. et al. 2019 [1].	NGS (10^−5^)	NTE NDMM pts (N = 737) - Dara-Rd arm - Rd arm	Longitudinal	Dara-Rd arm: 24.2% Rd arm: 7.3%	NA
Gay F. et al. 2019 [56]	MFC (10^−5^)	TE NDMM pts (N = 474) - KCd-ASCT-KCd (arm A, 159); - KRd-ASCT-KRd (arm B, 158); - 12 cycles of KRd (arm C, 157).	Longitudinal	Arm A: 42% Arm B: 58% Arm C: 54%	NA
Avet-Loiseau H. et al. 2019 [57]	MFC (10^−5^) and NGS (10^−6^)	TE NDMM pts (N = 1085) - Dara-VTd-ASCT-Dara-VTd - or VTd-ASCT-VTd	Post induction and post consolidation	Post induction MFC: Dara-VTd arm: 35%; VTd arm: 23% Post consolidation MFC: Dara-VTd arm: 64%; VTd arm: 44% Post consolidation NGS in evaluable patients: Dara-VTd arm: 39%; VTd arm 23%	NA
Voorhees P.M. et al. 2019 [4,5,58]	NGS (10^−5^)	TE NDMM pts (N = 104) Dara-VRd induction, ASCT and Dara-VRd consolidation	Longitudinal	Post induction: 15% Post consolidation: 44%	NA
Paiva B. et al. 2020 [23]	MFC (10^−4^, 10^−6^)	TE NDMM pts (N = 458) 6 VRd induction cycles, ASCT and 2 VRd consolidation cycles	CR patients, after induction, + 100 after ASCT, after consolidation	Post induction: 28% Post ASCT: 42% Post consolidation: 45%	PFS: 82% MRD neg vs. 50% MRD pos; 36 mo OS: 96% MRD neg vs. 88% MRD pos
Costa L.J. et al. 2019 [59]	NGS (<10^−5^)	TE NDMM pts (N = 81) Dara-KRd induction, ASCT, Dara-KRd consolidation	Longitudinal	Post induction: 40% Post ASCT: 73% Post consolidation: 82%	NA
Landgren O. et al. 2019 [60]	NGS (10^−5^)	TE and NTE NDMM pts (N = 24) 8 Dara-KRd cycles	After 8 cycles	75%	NA

**Abbreviations**. NDMM, newly diagnosed multiple myeloma; MRD, minimal residual disease; MFC, multiparameter flow cytometry; NGF, next-generation flow; NGS, next-generation sequencing; NTE; non-transplant-eligible; TE, transplant-eligible; N, number; V, bortezomib; M, melphalan; P, p, prednisone; R, lenalidomide; d, dexamethasone; Isa, isatuximab; K, carfilzomib; ASCT, autologous stem-cell transplantation; Dara, daratumumab; M, melphalan; C, cyclophosphamide; T, thalidomide; VGPR, very good partial response; CR; complete remission; TTP, time to progression; PFS, progression-free survival; NR, not reached; NA, not available; OS, overall survival; Neg, negative; pos, positive; mo, months.

**Table 3:** RRMM patients treated with novel agents: latest selected studies with MRD analysis.

Reference	MRD Technique (Sensitivity)	Study Population and Treatment	Time Point Assessment	MRD Rate	Outcome (mo)
Bahlis N. et al. 2018 [72]	NGS (10^−5^)	RRMM pts (N = 569) Dara-Rd vs. Rd	Longitudinal	30% vs. 5%	Median PFS, MRD neg: NR vs. 42; MRD pos: 30 vs. 16
Spencer A. et al. 2018 [74]	NGS (10^−5^)	RRMM pts (N = 498) Dara-Vd vs. Vd	Longitudinal	12% vs. 2%	Median PFS, MRD neg: NR vs. NR; MRD pos: NR vs. 16
Richardson P.G. et al. 2019 [71]	NGS (10^−5^)	RRMM pts (N = 307) Isa-Pd vs. Pd	Longitudinal	5% vs. 0%	NA
Usmani S. et al. 2019 [75]	NGS (10^−5^)	RRMM pts (N = 466) Dara-Kd vs. Kd	At 12 mo in pts in CR	13% vs. 1%	NA
Topp M.S. et. al. 2020 [76]	MFC (10^−4^)	RRMM pts (N = 42) AMG420	In ≥CR	50% of pts who received the MTD	NA
Raje N. et al. 2019 [73]	NGS (10^−4^) NGS (10^−5^) NGS (10^−6^)	RRMM pts (N = 33) Anti-BCMA CAR T bb2121	Post 1–3 mo after CAR T cell infusion	NGS (10^−4^): 100%; NGS (10^−5^): 94%; NGS (10^−6^): 19%	NA
Wang B.-Y. et al. 2019 [77]	MFC, 8 colors	RRMM pts (N = 5)	Longitudinal	68%	NA
Madduri D. et al. 2019 [78]	NGS (10^−4^) NGS (10^−5^) NGS (10^−6^)	RRMM pts (N = 29) Anti-BCMA CAR T JNJ-4528	Day +28	NGS (10^−4^): 18%; NGS (10^−5^): 29%; NGS (10^−6^): 53%	NA

**Abbreviations**. RRMM, relapsed/refractory multiple myeloma; MRD, minimal residual disease; NGS, next-generation sequencing; MFC, multiparameter flow cytometry; N, number; Dara, daratumumab; R, lenalidomide; d, dexamethasone; V, bortezomib; Isa, isatuximab; P, pomalidomide; K, carfilzomib; CR, complete response; pts, patients; MTD, maximum tolerated dose; PFS, progression-free survival; neg, negative; pos, positive; NR, not reached; NA, not available; mo, months.
