# Erratum: Mina, R.; et al. Minimal Residual Disease in Multiple Myeloma: State of the Art and Future Perspectives. *J. Clin. Med.* 2020, *9*, 2142

**DOI:** 10.3390/jcm9082630

**Published:** 2020-08-13

**Authors:** Roberto Mina, Stefania Oliva, Mario Boccadoro

**Affiliations:** Myeloma Unit, Division of Hematology, University of Torino, Azienda Ospedaliero-Universitaria Città della Salute e della Scienza di Torino, via Genova 3, 10126 Torino, Italy; stefania.olivamolinet@gmail.com (S.O.); mario.boccadoro@unito.it (M.B.)

The authors sincerely apologize for the inaccuracies made during the revision that a product line has been associated with the wrong company. They wish to make the following corrections to the previous paper [1]. 

On page 2 of the published paper (Section 2.1. *MRD in the Bone Marrow*), the authors would like to replace the inaccurate sentence:

Other non-commercial NGS technologies are under investigation: the LymphoTrack^®^ assay (Invitrogen, US-MA) has been recently validated in a phase II study [2], and the EuroClonality-NGS Consortium (an international group of 21 academic laboratories experienced in NGS) has recently validated IG/TR NGS assays and a bioinformatic tool for an academic study on MRD [3].

With the following sentence:

Other non-commercial NGS technologies are under investigation: the LymphoTrack^®^ assay (Invivoscribe, Inc., US-CA) has been recently used to establish IGH chain clonality for NGS characterization of B-Cell and plasma cell neoplasms [4]. Martinez-Lopez et al. validated an “in-house deep-sequencing” method in a phase II study by using standardized primers developed by the Biomed-2 Concerted Action, in order to amplify all IGH or IGK sequences [2]. Furthermore, the EuroClonality-NGS Consortium (an international group of 21 academic laboratories experienced in NGS) has recently validated IG/TR NGS assays and a bioinformatic tool for an academic study on MRD [3].

The published version will be updated online on the article webpage, with a reference to this Erratum.
